# *Collagen type VI α5* gene variations may predict the risk of lung cancer development in Chinese Han population

**DOI:** 10.1038/s41598-020-61614-x

**Published:** 2020-03-19

**Authors:** Ying Duan, Gaowen Liu, Yao Sun, Jiamin Wu, Zichao Xiong, Tianbo Jin, Mingwei Chen

**Affiliations:** 1 Department of Respiratory Medicine, The First Affiliated Hospital of School of Medicine of Xi’an Jiaotong University, Xi’an, Shaanxi 710061 China; 2 Xianyang Central hospital, Xianyang, Shaanxi 712000 China; 3 Key Laboratory of Resource Biology and Biotechnology in Western China, Ministry of Education. School of Medicine, Northwest University, Xi’an, Shaanxi 710069 China

**Keywords:** Genetics, Cancer genetics

## Abstract

The abundant expression of *collagen type VI α5* (*COL6A5*) exists in lung tissue, and its role in lung cancer is still unknown. We performed a genetic association study with an attempt to detect the relationships between single nucleotide polymorphisms (SNPs) in *COL6A5* and lung cancer predisposition in Chinese Han population. We finally selected six tag-SNPs to determine their genotypes among 510 lung cancer patients and 495 healthy controls with the MassARRAY platform. The associations of SNPs and lung cancer risk were estimated by logistic regression method with adjustment for confounding factors. Two available databases were used for gene expression and prognosis analysis. *COL6A5* rs13062453, rs1497305, and rs77123808 were significantly associated with the risk of lung cancer in the whole population or stratified subgroups (*p* < 0.05). Among them, *COL6A5* rs13062453 and rs1497305 were also linked to the susceptibility of lung adenocarcinoma. Additionally, rs1497305 was found to be strongly related to the TNM staging under five genetic models (*p* < 0.05). Results from databases suggested the important role of *COL6A5* in lung cancer development. *COL6A5* polymorphisms rs13062453, rs1497305 and rs77123808 were associated with lung cancer risk in Chinese Han population. These findings first yield new insight of *COL6A5* in lung cancer.

## Introduction

The incidence and mortality rates of lung cancer rank first worldwide, with 2,093,876 (11.6% of total cancer patients) new cases and 1,761,007 (18.4% of total cancer deaths) deaths occurred globally in 2018^1^. Considering the sex-distribution difference, lung cancer is the leading cause of cancer incidence (1,368,524 new cases) and death (1,184,947 deaths) among males, accounting for approximately 14.4% new cases and 22.0% deaths respectively, and the third prevalent cancer (725,352 new cases, 8.4%) with second fatality rate (576,060 deaths, 13.8%) among females^1^. The morbidity and mortality of lung cancer is very high in China, even worse, continue rising in recent years^2^. Previous epidemiological studies have reported a dramatical increase of crude incidence of lung cancer among Chinese from 2000 to 2014, which reached 57.13 per 100,000 in 2014 (74.31 per 100,000 in males and 39.08 per 100,000 in females)^2,3^. In the same year, the estimated mortality rate of lung cancer deaths is 45.80 per 100,000 (61.10 per 100,000 in males and 29.71 per 100,000 in females)^3^. Multiple etiological analyses have found that external risk factors such as smoking, air pollution and occupational exposure contribute to the lung tumorigenesis^4–6^. Moreover, researchers have emphasized that genetic factors play critical roles in the development of lung cancer as well. Nowadays, accumulating evidence has elucidated that genetic polymorphisms are risk markers that can mediate individual susceptibility to lung cancer^7–11^. These findings also provide multiple promising indicators that pave the road for individual cancer risk assessment. Therefore, exploring these novel factors is a vital step which not only reveal the underlying genetic mechanisms of lung cancer, but facilitate cancer risk evaluation among populations with diverse genetic backgrounds.

Collagen type VI has been identified as a extracellular matrix protein with functional role and ubiquitous distribution in numerous connective tissues, where it forms microfibrils organized into extend microfilamentous network^12^. It has been well established for a long time that collagen VI is comprised of three genetically distinct α-chains, namely collagen VI α1, α2 and α3, which is encoded by *COL6A1*, *COL6A2*, and *COL6A3* respectively^13^. Subsequently, three new collagen VI chains were identified, and designated α4, α5 and α6 chains that are encoded by distinct genes, *COL6A4*, *COL6A5*, and *COL6A6*^14^. The aberrant expression of collagen VI genes has been reported in several malignant tumors, suggesting the potential effects of collagen VI in cancer development^15–17^. According to previous publications, the interaction between collagen VI and other components will lead to the remodeling of extracellular matrix, and stimulate the signaling pathway implicated in cell proliferation, migration, differentiation and apoptosis^18–21^. Collagen VI exerts important functions in maintaining the integrity of lung tissue^22^. And *COL6A5* (Collagen type VI α5), which is also known as *COL29A1*, was highly expressed in lung tissue as well as skin, whereas less is reported on lung cancer^23^. Accordingly, we hypothesized that the single nucleotide polymorphisms (SNPs) in *COL6A5* might be associated with lung cancer risk, and performed a genetic association study to investigate the relationships between the *COL6A5* variations and lung cancer predisposition among Chinese Han population.

## Materials and Methods

### Ethics statement and consent to subjects

This study was performed with the approval of the ethics committee of The First Affiliated Hospital of School of Medicine of Xi’an Jiaotong University and all the procedures conformed to the 1964 Helsinki declaration and its later amendments. All the subjects were informed verbally and in writting of the protocols in this work. Signed informed consent was obtained prior to participation.

### Study participants

The present study was carried out based on two groups of sample: 510 patients with diagnosed lung cancer and 495 healthy individuals. All the participants were recruited from Shaanxi Provincial Cancer Hospital. Cases were hospitalized from 2015 to 2018 and histopathological diagnosed as primary lung carcinoma by at least two experienced pathologists. Electronic bronchoscopy, percutaneous lung puncture, sputum exfoliation and pleural effusion cytology examination were supplementary methods for diagnosis. Patients with other cancers, cancer history or chronic diseases of respiratory system (for example: chronic obstructive pulmonary disease and asthma), and had radiation or chemical therapy were excluded from this work. The clinical information and basic characteristics of the patients were recorded by physicians. During the same time, healthy subjects were randomly enrolled when they were attending the routine physical examination at the same hospital. The exclusion criteria of the control group were as follows: 1) previous malignancy history; 2) chronic respiratory diseases and autoimmune disorders; 3) family history of pulmonary tumor or genetic diseases. The eligible controls and cases were hereditarily and unrelated Chinese Han individuals from northwest China. The numbers of male and female were matched in 1:1 fashion between cases and controls. During the recruitment, we randomly screened the samples according to the inclusion and exclusion criteria and tried our best to avoid the population stratification.

### SNP selection and genotyping

**Figure 1:**
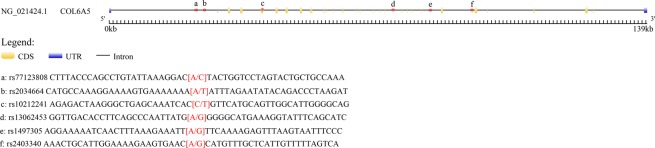
*COL6A5* gene schematic representation. The specific location of each SNP is marked by asterisk. Minimum unit of the measure is 1 kb. The flanking sequences of SNPs were provided by dbSNP database (http://www.bioinfo.org.cn/relative/dbSNP%20Home%20Page.htm).

Searching the 1000 Genomes website (http://www.internationalgenome.org/), we downloaded the data of *COL6A5* variations in CHB (Chinese Han Beijing) and then inputted them into haploview 4.2 software. The SNPs whose minor allele frequency (MAF) > 0.05, Hardy-Weinberg equilibrium (HWE) > 0.01 and r^2^ > 0.8 were selected for linkage disequilibrium block construction. Subsequently, we obtained several tag-SNPs which could represent the polymorphisms of the strong linkage regions. Ultimately, six *COL6A5* polymorphisms, rs77123808, rs2034664, rs10212241, rs13062453, rs1497305, and rs2403340 were selected for genotyping. The schematic representation of *COL6A5* gene was performed by Gene Structure Display Server version 2.0 (http://gsds.cbi.pku.edu.cn/), and specific location of each SNP is marked in Fig. 1. The variants were further searched in dbSNP database (http://www.bioinfo.org.cn/relative/dbSNP%20Home%20Page.htm) to acquire detailed genetic information. A volume of 3–5 ml peripheral blood was donated from the participants and stored in ethylene diamine tetraacetic acid (EDTA) anticoagulant tubes at −80 °C. GoldMag-Mini Whole Blood Genomic DNA Purification Kit (GoldMag Co. Ltd, Xi’an City, China) was used for genomic DNA extraction from each sample according to the standard protocol. The purified and concentrated DNA was evaluated by NanoDrop spectrophotometer (Thermo Scientific, Waltham, Massachusetts, USA) at wavelength of 260 nm and 280 nm. The qualified DNA was used as the template for the primary polymerase chain reaction (PCR), and followed by a unique base extension. Using the Agena MassARRY method (Agena Bioscience, San Diego, CA, USA), the genotypes of the six variants were assayed with products of the last PCR step. The genotype data was read out by the Agena Bioscience TYPER software, version 4.0.

### Statistical analysis

The distribution differences of the age and gender among cases and controls were assessed by independent samples *t*-test and Pearson’s chi-squared test, respectively. Genotype frequency of the variations in the two study groups were compared with Pearson’s chi-squared test. HWE *p* value was also calculated in controls using Pearson’s chi-squared test. Furthermore, logistic regression analysis with adjustment was conducted to estimate the associations of the candidate SNPs with lung cancer susceptibility in genetic models (allele model, genotype model, dominant model, recessive model, and additive model) via available odds ratio (OR) values and 95% confidence intervals (CIs). The minor allele of each variation was hypothesized as the risk factor in our research. In statistical analysis, *p* = 0.05 was considered significant threshold. Stratified analyses by age, gender, pathological type, and TNM stage were carried out as well. SPSS version 20.0 (IBM SPSS, Inc., Chicago, IL, USA) and PLINK package, version 2.1.7 were used for basic characteristic statistics and risk evaluation.

### Expression and prognosis analyses in databases

In order to further investigate the role of *COL6A5* gene in lung tumorigenesis, we performed a large sample-based expression detection in Gene Expression Profiling Interactive Analysis (GEPIA) database (http://gepia.cancer-pku.cn/) and overall survival analysis in Kaplan-Meier Plotter (Lung cancer, http://kmplot.com/analysis/index.php?p=service&cancer=lung). The expression levels of *COL6A5* were determined in lung cancer (including lung adenocarcinoma and lung squamous cell carcinoma) tissues and normal samples from healthy individuals. The prognostic differences between patients with high and low expression of *COL6A5* were assessed by hazard ratio (HR) and log-rank *p* value.

## Results

### Expression and prognosis patterns of *COL6A5* gene

**Figure 2:**
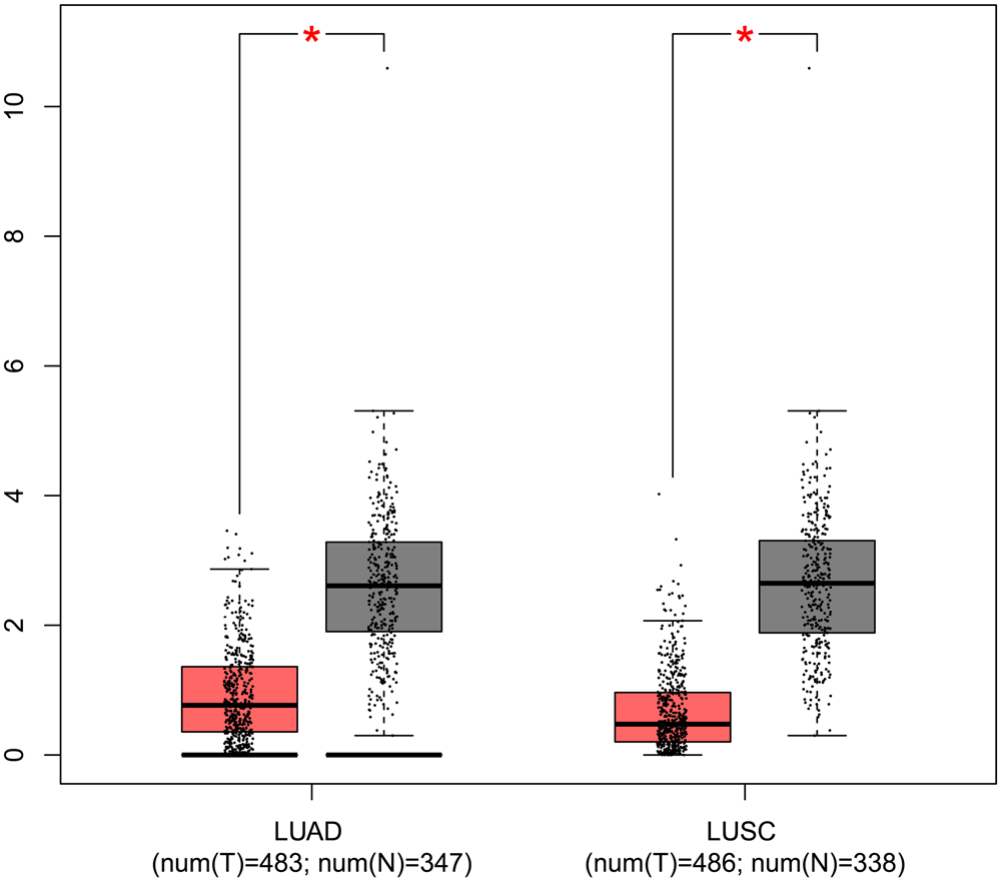
Comparison of the *COL6A5* gene expression in lung adenocarcinoma (LUAD, N = 483), squamous cell carcinoma (LUSC, N = 486) tumors and healthy tissues (LUAD normal tissues: N = 347; LUSC normal tissues: N = 338) in GEPIA database. The expression of *COL6A5* gene was down-regulated in both lung cancer types. *p < 0.05.

**Figure 3:**
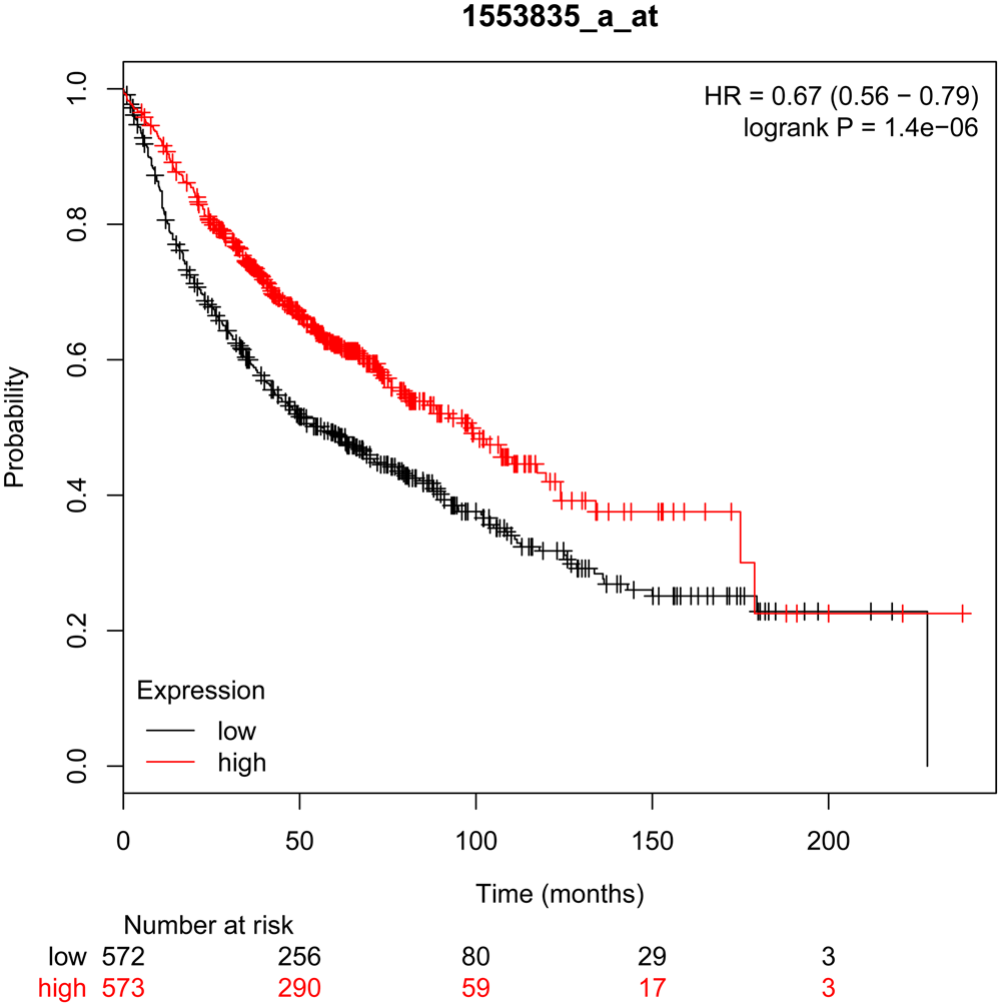
Low expression of *COL6A5* gene is associated with poor overall survival in lung cancer patients. The Kaplan-Meier curves presented the overall survival of lung cancer patients with high (red) and low (black) *COL6A5* expression levels and generated by Kaplan–Meier method.

With the GEPIA database, we detected the expression levels of *COL6A5* in lung tumors and normal tissues. Overall, *COL6A5* was down-regulated in both lung adenocarcinoma and lung squamous cell carcinoma (Fig. 2). Subsequently, prognosis analysis indicated that patients with low expression of *COL6A5* had worse overall survival (Fig. 3, HR = 0.67, 95% CI = 0.56–0.79, *p* < 0.001).

### Study subjects

The basic characteristics of the 510 lung cancer patients and 495 healthy individuals were compared and showed in Table 1. The case group consisted of 355 males and 155 females, with a mean age of 60.78 ± 9.96. Moreover, pathological type, TNM classification and lymph node metastasis status of the most lung cancer patients were also collected and involved in further stratified analysis. As for the controls, 346 males and 149 females were enrolled, which were matched to the cases in an approximately 1:1 fashion (*p* = 0.920). The mean age of the healthy subjects was 61.94 ± 7.72 years in this study.

**Table 1:** Demographic information of the lung cancer cases and healthy controls in this study.

Variables	Lung Cancer Cases (N = 510)	Healthy Controls (N = 495)	*p* value
Age (years, mean ± SD)	60.78 ± 9.96	61.94 ± 7.72	0.038^a^
**Gender**			
Male	355	346	0.920^b^
Female	155	149	
**Pathological Type**			
Lung Adenocarcinoma	188		
Squamous Cell Carcinoma	120		
Others	202		
**TNM Stage**			
I+II	84		
III+IV	261		
Absence	165		
**Lymph Node Metastasis**			
Positive	215		
Negative	84		
Absence	211		

^a^*p* value was calculated with independent samples *t*-test.

^b^*p* value was calculated with Pearson’s chi-squared test.

### Basic information of the candidate SNPs

Specific information and minor allele frequency of the six candidate SNPs are listed in Table 2, including rs77123808 (C < A), rs2034664 (T < A), rs10212241 (T < C), rs13062453 (A < G), rs1497305 (A < G), and rs2403340 (A < G). The call rate for each SNP was over 99% in the current study. All the selected variants are located in *COL6A5*, chromosome 3, with MAF > 0.05 in both cases and controls. From Table 2, we found that the frequency of minor allele “T” of rs10212241 (0.438 in cases and 0.438 in controls) and “A” of rs2403340 (0.175 in cases and 0.174 in controls) were similar among lung cancer patients and healthy subjects. Genotype distributions of the six *COL6A5* variants in lung cancer cases and healthy controls are showed in Supplementary Table S1. Pearson’s chi-squared test *p* value of rs1497305 indicated that there was difference in genotype distribution between the two groups (*p* = 0.020). In control group, the HWE *p* value of each variant was calculated (rs77123808: 1.00. rs2034664: 0.04, rs10212241: 1.00, rs13062453: 0.15, rs1497305: 0.09, rs2403340: 0.04). Because of the lower MAF in our population, rs2034664 and rs2403340 exhibited marginal HWE *p* values and were excluded from further analysis.

**Table 2:** Descriptive information of the selected SNPs in *COL6A5*.

SNP	Chromosome	Position	Functional Consequence	Alleles A < B	Minor Allele Frequency		Call Rate
					Case	Control	
rs77123808	Chr 3	130368023	intron variant	C < A	0.338	0.356	100.00%
rs2034664	Chr 3	130370233	intron variant	T < A	0.183	0.170	99.90%
rs10212241	Chr 3	130385219	transcript variant (synonymous codon)	T < C	0.438	0.438	99.90%
rs13062453	Chr 3	130419022	intron variant	A < G	0.365	0.328	99.80%
rs1497305	Chr 3	130428544	intron variant	A < G	0.289	0.276	99.30%
rs2403340	Chr 3	130439499	intron variant	A < G	0.175	0.174	99.90%

SNP: Single nucleotide polymorphism; Chr: Chromosome.

### Association analysis of *COL6A5* variants with lung cancer risk

We first investigated the relationships between rs77123808, rs10212241, rs13062453, rs1497305 and lung cancer risk in the Chinese Han population. As presented in Table 3, the heterozygous “GA” genotype of rs13062453 was associated with an enhanced risk of lung cancer when compared with the homozygous “GG” genotype (adjusted OR = 1.33, 95% CI = 1.02–1.74, *p* = 0.036, power = 61.77%). Genetic model analysis revealed statistical significance of this variants in dominant model as well (adjusted OR = 1.30, 95% CI = 1.01–1.68, *p* = 0.039, power = 54.43%; Supplementary Table S2). Likewise, remarkable correlation also existed between the “GA” genotype of rs1497305 and increased lung cancer susceptibility among Chinese Han individuals according to an adjusted OR of 1.37 (95% CI = 1.05–1.78, power = 69.11%) and significant *p* value of 0.019 (Table 3). However, rs77123808 and rs10212241did not show any associations with lung cancer in our study cohort (*p* > 0.05).

**Table 3:** Evaluation of the correlation between *COL6A5* variants and lung cancer susceptibility among Chinese Han population.

SNP	Allele/Genotype	Control N (%)	Case N (%)	OR (95% CI)	*p* value
rs77123808	A	638 (64.44%)	675 (66.18%)	1.00	0.415
	C	352 (35.56%)	345 (33.82%)	0.93 (0.77–1.11)	
	AA	205 (41.41%)	221 (43.33%)	1.00	
	AC	228 (46.06%)	233 (45.69%)	0.96 (0.73–1.24)	0.735
	CC	62 (12.53%)	56 (10.98%)	0.85 (0.56–1.27)	0.423
rs10212241	C	555 (56.17%)	573 (56.18%)	1.00	0.999
	T	433 (43.83%)	447 (43.82%)	1.00 (0.84–1.19)	
	CC	156 (31.58%)	163 (31.96%)	1.00	
	CT	243 (49.19%)	247 (48.43%)	0.97 (0.73–1.29)	0.858
	TT	95 (19.23%)	100 (19.61%)	1.01 (0.71–1.45)	0.950
rs13062453	G	664 (67.21%)	646 (63.46%)	1.00	0.078
	A	324 (32.79%)	372 (36.54%)	1.18 (0.98–1.42)	
	GG	230 (46.56%)	203 (39.88%)	1.00	
	GA	204 (41.30%)	240 (47.15%)	**1.33 (1.02–1.74)**	0.036*
	AA	60 (12.15%)	66 (12.97%)	1.21 (0.81–1.81)	0.346
rs1497305	G	710 (72.45%)	722 (71.06%)	1.00	0.492
	A	270 (27.55%)	294 (28.94%)	1.07 (0.88–1.30)	
	GG	265 (54.08%)	247 (48.62%)	1.00	
	GA	180 (36.73%)	228 (44.88%)	**1.37 (1.05–1.78)**	0.019*
	AA	45 (9.18%)	33 (6.50%)	0.76 (0.47–1.24)	0.269

SNP: Single nucleotide polymorphism; OR: Odds ratio; 95% CI: 95% confidence interval.

The OR and 95% CI were calculated by logistic regression analysis.

*p* value was obtained by Wald test with adjustment for age and gender.

*Statistically significant results are in bold (*p* < 0.05).

### Association analysis in stratified subgroups

Furthermore, stratified analysis was performed in order to explore the effects of these SNPs on lung cancer in different subgroups. First, the correlations between the genetic variations and lung cancer risk were evaluated among people with different age and gender, and the significant *COL6A5* variants were summarized in Table 4 (allele and genotype analysis) and Supplementary Table S3 (Genetic model analysis). Among the individuals aged younger than 61 years, the rs1497305 “GA” carriers harbored an increased risk of lung cancer when compared with “GG” carriers (adjusted OR = 1.59, 95% CI = 1.08–2.34, *p* = 0.019). In females, rs13062453 and rs1497305 polymorphisms elevated the risk of lung cancer according to the genotype and dominant model (rs13062453: “GA” genotype adjusted OR = 1.75, 95% CI = 1.07–2.86, *p* = 0.027, dominant model adjusted OR = 1.68, 95% CI = 1.05–2.86, *p* = 0.030; rs1497305: “AA” genotype adjusted OR = 1.79, 95% CI = 1.10–2.90, *p* = 0.019, dominant model adjusted OR = 1.59, 95% CI = 1.01–2.52, *p* = 0.046), whereas rs77123808 reduced the predisposition to lung tumorigenesis (“AC” adjusted OR = 0.51, 95% CI = 0.31–0.83, *p* = 0.006, dominant model adjusted OR = 0.58, 95% CI = 0.37–0.92, *p* = 0.021). The significant associations could not be detected for other candidate SNPs and in group older than 61 years or males (*p* > 0.05). Second, we examinated the associations of *COL6A5* SNPs with two common pathological types of lung cancer, namely lung adenocarcinoma and squamous cell carcinoma. As showed in Table 5, the “GA” genotype of rs13062453 and rs1497305 wasfound to be correlated to the increased risk of lung adenocarcinoma (rs13062453: adjusted OR = 1.46, 95% CI = 1.01–2.11, *p* = 0.045; rs1497305: adjusted OR = 1.76, 95% CI = 1.23–2.51, *p* = 0.002). Rs1497305 also showed obvious evidence in correlation to lung adenocarcinoma susceptibility in dominant model (adjusted OR = 1.51, 95% CI = 1.07–2.13, *p* = 0.018) and recessive model (adjusted OR = 0.45, 95% CI = 0.22–0.96, *p* = 0.038) (Supplementary Table S4). However, analysis on squamous cell carcinoma did not uncover any significant SNPs. Lastly, the contributions of *COL6A5* variants on TNM staging and lymph node metastasis were demonstrated. Notably, pronounced results of the polymorphism rs1497305 still survived in all statistical analysis for TNM staging even after adjustment, which suggested the importance of this SNP in lung cancer progression (*p* < 0.05, Table 6 and Supplementary Table S5). Unexpectedly, there were no significant findings between all the selected SNPs and lymph node metastasis (*p* > 0.05, Supplementary Tables S6 and S7).

**Table 4:** Significant *COL6A5* variants associated with lung cancer susceptibility in stratified analysis for age or gender.

SNP	Allele/Genotype	> 61				<61			
		Control N (%)	Case N (%)	OR (95% CI)	*p* value	Control N (%)	Case N (%)	OR (95% CI)	*p* value
rs1497305	G	395 (73.42%)	355 (73.35%)	1.00	0.979	315 (71.27%)	367 (68.98%)	1.00	0.439
	A	143 (26.58%)	129 (26.65%)	1.00 (0.76–1.33)		127 (28.73%)	165 (31.02%)	1.12 (0.85–1.47)	
	GG	146 (54.28%)	124 (51.24%)	1.00		119 (53.85%)	123 (46.24%)	1.00	
	GA	103 (38.29%)	107 (44.21%)	1.19 (0.82–1.71)	0.360	77 (34.84%)	121 (45.49%)	**1.59 (1.08–2.34)**	0.019*
	AA	20 (7.43%)	11 (4.55%)	0.61 (0.28–1.34)	0.217	25 (11.31%)	22 (8.27%)	0.83 (0.44–1.56)	0.559
**SNP**	**Allele/Genotype**	**Male**				**Female**			
		**Control N (%)**	**Case N (%)**	**OR (95% CI)**	***p*** **value**	**Control N (%)**	**Case N (%)**	**OR (95% CI)**	***p*** **value**
rs77123808	A	450 (65.03%)	464 (65.35%)	1.00	0.899	188 (63.09%)	209 (67.86%)	1.00	0.217
	C	242 (34.97%)	246 (34.65%)	0.99 (0.79–1.23)		110 (36.91%)	99 (32.14%)	0.81 (0.58–1.13)	
	AA	152 (43.93%)	145 (40.85%)	1.00		53 (35.57%)	75 (48.70%)	1.00	
	AC	146 (42.20%)	174 (49.01%)	1.28 (0.93–1.76)	0.132	82 (55.03%)	59 (38.31%)	**0.51 (0.31–0.83)**	0.006*
	CC	48 (13.87%)	36 (10.14%)	0.81 (0.49–1.32)	0.396	14 (9.40%)	20 (12.99%)	1.01 (0.47–2.18)	0.980
rs13062453	G	468 (67.63%)	461 (65.11%)	1.00	0.319	196 (66.22%)	184 (59.74%)	1.00	0.100
	A	224 (32.37%)	247 (34.89%)	1.12 (0.90–1.40)		100 (33.78%)	124 (40.26%)	1.32 (0.95–1.84)	
	GG	163 (47.11%)	152 (42.94%)	1.00		67 (45.27%)	51 (33.12%)	1.00	
	GA	142 (41.04%)	157 (44.35%)	1.20 (0.87–1.65)	0.261	62 (41.89%)	82 (53.25%)	**1.75 (1.07–2.86)**	0.027*
	AA	41 (11.85%)	45 (12.71%)	1.12 (0.69–1.81)	0.651	19 (12.84%)	21 (13.64%)	1.46 (0.71–3.00)	0.305
rs1497305	G	503 (72.90%)	515 (72.95%)	1.00	0.984	207 (71.38%)	206 (66.88%)	1.00	0.235
	A	187 (27.10%)	191 (27.05%)	1.00 (0.79–1.26)		83 (28.62%)	102 (33.12%)	1.24 (0.87–1.75)	
	GG	187 (54.20%)	182 (51.56%)	1.00		78 (53.79%)	65 (42.21%)	1.00	
	GA	129 (37.39%)	151 (42.78%)	1.22 (0.89–1.66)	0.223	51 (35.17%)	76 (49.35%)	0.98 (0.44–2.18)	0.951
	AA	29 (8.41%)	20 (5.67%)	0.66 (0.36–1.21)	0.180	16 (11.03%)	13 (8.44%)	**1.79 (1.10–2.90)**	0.019*

SNP: Single nucleotide polymorphism; OR: Odds ratio; 95% CI: 95% confidence interval.

The OR and 95% CI were calculated by logistic regression analysis.

*p* value was obtained by Wald test with adjustment for age and gender.

*Statistically significant results are in bold (*p* < 0.05).

**Table 5:** Significant *COL6A5* variants in stratified analysis for pathological type.

SNP	Allele/Genotype	Control (N = 495)	Lung Adenocarcinoma			Squamous Cell Carcinoma		
			Case (N = 188)	OR (95% CI)	*p*^a^-value	Case (N = 120)	OR (95% CI)	*p*^a^-value
rs13062453	G	664 (67.21%)	233 (61.97%)	1.00	0.068	157 (65.97%)	1.00	0.715
	A	324 (32.79%)	143 (38.03%)	1.26 (0.98–1.61)		81 (34.03%)	1.06 (0.78–1.43)	
	GG	230 (46.56%)	70 (37.23%)	1.00		52 (43.70%)	1.00	
	GA	204 (41.30%)	93 (49.47%)	**1.46 (1.01–2.11)**	0.045*	53 (44.54%)	1.18 (0.76–1.83)	0.456
	AA	60 (12.15%)	25 (13.30%)	1.27 (0.74–2.20)	0.391	14 (11.76%)	1.07 (0.55–2.09)	0.847
rs1497305	G	710 (72.45%)	261 (69.41%)	1.00	0.267	168 (70.59%)	1.00	0.566
	A	270 (27.55%)	115 (30.59%)	1.16 (0.89–1.50)		70 (29.41%)	1.10 (0.80–1.50)	
	GG	265 (54.08%)	93 (46.73%)	1.00		59 (49.58%)	1.00	
	GA	180 (36.73%)	97 (48.74%)	**1.76 (1.23–2.51)**	0.002*	50 (42.02%)	1.24 (0.81–1.91)	0.323
	AA	45 (9.18%)	9 (4.52%)	0.59 (0.27–1.27)	0.180	10 (8.40%)	1.08 (0.50–2.31)	0.848

SNP: Single nucleotide polymorphism; OR: Odds ratio; 95% CI: 95% confidence interval.

The OR and 95% CI were calculated by logistic regression analysis.

*p* value was obtained by Wald test with adjustment for age and gender.

*Statistically significant results are in bold (*p* < 0.05**)**.

**Table 6:** Significant *COL6A5* variants associated with TNM staging of lung cancer among Chinese Han population.

SNP	Allele/Genotype	Control N (%)	Case N (%)	OR (95% CI)	*p* value
rs1497305	G	105 (62.50%)	377 (72.78%)	1.00	0.011*
	A	63 (37.50%)	141 (27.22%)	**0.62 (0.43–0.90)**	
	GG	32 (38.10%)	134 (51.74%)	1.00	
	GA	41 (48.81%)	109 (42.08%)	0.63 (0.37–1.07)	0.089
	AA	11 (13.10%)	16 (6.18%)	**0.32 (0.13–0.76)**	0.010*

SNP: Single nucleotide polymorphism; OR: Odds ratio; 95% CI: 95% confidence interval.

The OR and 95% CI were calculated by logistic regression analysis.

*p* value was obtained by Wald test with adjustment for age and gender.

*Statistically significant results are in bold (*p* < 0.05).

## Discussion

The current study evaluated the correlations between polymorphisms in *COL6A5* gene and lung cancer susceptibility among Chinese Han individuals. We found that *COL6A5* rs13062453, rs1497305, and rs77123808 were associated with the risk of lung cancer in Chinese Han population. Additionally, *COL6A5* rs1497305 was a potential predictor for TNM staging. Basing on the expression patterns presented by databases, we found a down-regulation of *COL6A5* in lung tumors when compared with the normal tissues. The low expression level was linked to the worse overall survival of lung cancer patients. These results preliminary elucidated the potential genetic determinants in *COL6A5* of lung cancer, and first uncovered the involvement of *COL6A5* gene in lung carcinogenesis.

Collage family consists of 28 types of extracellular matrix proteins with α chains, which can facilitate cell growth and maintain the mechanical elasticity of connective tissue^24^. Collagen type VI plays a prominent role in both tumorigenesis and metastasis^25^. Collagen type VI α1, α2 and α3 are three major extracellular matrix protein of collagen type VI subgroup, which can promote tumor growth^25^. Collagen type VI α4, α5 and α6 are three members that are identified lately, with a N-terminal region containing seven vWF-A modules, a collagen triple helical region, and a C-terminal region made of two or three vWF-A modules as well as specific sequence^14,26^. However, it is largely unknown how collagen type VI α4, α5 and α6 are implicated in tumor development. COL6A6 has been reported to be down-regulated in breast cancer^27–29^. Significant down-regulation of COL6A5 has been proved in esophageal squamous cell carcinoma^30^ and fibrous epulis^31^. Wang and the colleagues has found that collagen type VI may interacts with ROBO2 in esophageal squamous cell carcinoma development, and ROBO2 serves as a tumor suppressor during malignant process^32^. Collagen type VI serves as an indispensable protein in extracellular matrix, and mainly deposited in lung fibrosis^33^. Pulmonary fibrosis, combined with emphysema, has been fully demonstrated to be strongly associated with the development of lung cancer^34,35^. However, the characteristics of *COL6A5* gene in lung cancer remains incompletely understood. Bioinformatics analyses showed a decrease of *COL6A5* level in lung tumor, and the low expression resulted in poor prognosis. We thus speculated that *COL6A5* functions as a tumor suppressor gene in oncogenesis of lung. More convincing experimental evidence is needed to confirm these findings.

Single nucleotide substitutions in genes have been validated to be the risk factors in diverse diseases, which are proposed as predictors for individual risk assessment. Susceptible SNPs of *COL6A5* have been identified and demonstrated in familial neuropathic chronic itch in previous research^36^. Eight variants located in *COL6A5* gene have been demonstrated to be related to the susceptibility to atopic dermatitis among Europeans^37^. A longitudinal exome-wide association study performed among Japanese has showed that *COL6A5* SNP rs11917356 was significantly correlated with hypertension risk through affect systolic blood pressure^38^. Furthermore, it has been reported that variations in *COL6A5* might play causal roles for asthma together with environmental exposures^39,40^. But a recent study has not found any significant relationships between *COL6A5* variants and asthma or chronic obstructive pulmonary disease in German^41^. In our study, *COL6A5* polymorphisms rs13062453, rs1497305, and rs77123808 were genetic polymorphic markers that confer susceptibility to lung cancer among Chinese Han individuals. As all the significant SNPs were resided in the intron region of *COL6A5* gene, which possesses regulatory functions in pre-mRNA processing^42^, we considered that alternation at these polymorphic sites might modulate the expression efficiency of *COL6A5* mRNA, contributing to the abnormal pattern of *COL6A5* and thereby influencing the individual susceptibility to lung cancer. Additionally, *COL6A5* rs1497305 was found to be associated with TNM staging, which harbors the potential to become new target for lung cancer clinical stage evaluation in future.

There are several limitations in our research that should be acknowledged. First, all the cases and controls are recruited from one hospital, and the possible selection bias may exist. The random subject selection could help us reduce the selection bias to some degree. Second, the expression detection and prognosis analysis of *COL6A5* gene were carried out by databases, which should be verified with well-designed experimental study. Third, the underlying mechanism of the promising SNPs and *COL6A5* gene in lung cancer development is still unclear, thus suggesting the nest step for the further research.

In summary, this is the first research that discussed the relationships between *COL6A5* polymorphisms and lung cancer risk. We uncovered significant variants that were associated with lung cancer susceptibility in Chinese Han population. These findings also provided supporting evidence for the involvement of *COL6A5* gene in the occurrence of lung cancer.

## Supplementary information

Supplementary information.

## Data Availability

The data generated or analyzed during this study are available from the corresponding author on reasonable request.
