# Supplementary material for: Collagen type VI α5 gene variations may predict the risk of lung cancer development in Chinese Han population
Source: Sci Rep. 2020 Mar 19;10:5010. doi: 10.1038/s41598-020-61614-x (PMC7081318; doi:10.1038/s41598-020-61614-x)
Supplement: Supplementary file 1 — Supplementary information. [file 41598_2020_61614_MOESM1_ESM.docx]

***Collagen type VI α5* gene variations may predict the risk of lung cancer development in Chinese Han population**

Ying Duan^1^, Gaowen Liu^2^, Yao Sun^3^, Jiamin Wu^3^, Zichao Xiong^3^, Tianbo Jin^3^, Mingwei Chen^1,#^

1. Department of Respiratory Medicine, The First Affiliated Hospital of School of Medicine of Xi’an Jiaotong University, Xi’an, Shaanxi 710061, China

2. Xianyang Central hospital, Xianyang, Shaanxi, 712000, China

3. Key Laboratory of Resource Biology and Biotechnology in Western China (Northwest University), Ministry of Education, Xi’an, Shaanxi 710069, China

#Corresponding Author:

**Mingwei Chen** M.D. & Professor

Tel/Fax: 13088988001

E-mail: mingwei__chen@163.com

Address: #277 Yanta West Road, Xi'an710061, Shaanxi, China

Supplementary Table S1 Genotype distribution of the six SNPs in lung cancer case and healthy control groups

| SNP | Genotype | Control | Case | *p* value |
| --- | --- | --- | --- | --- |
| rs77123808 | AA | 205 | 221 | 0.692 |
|  | AC | 228 | 233 |  |
|  | CC | 62 | 56 |  |
| rs2034664 | AA | 347 | 339 | 0.173 |
|  | AT | 126 | 155 |  |
|  | TT | 21 | 16 |  |
| rs10212241 | CC | 156 | 163 | 0.971 |
|  | CT | 243 | 247 |  |
|  | TT | 95 | 100 |  |
| rs13062453 | GG | 230 | 203 | 0.097 |
|  | GA | 204 | 240 |  |
|  | AA | 60 |  |  |
| rs1497305 | GG | 265 | 247 | **0.020*** |
|  | GA | 180 | 228 |  |
|  | AA | 45 | 33 |  |
| rs2403340 | GG | 344 | 347 | 0.365 |
|  | GA | 128 | 147 |  |
|  | AA | 22 | 16 |  |

SNP: Single nucleotide polymorphism.

*p* value was obtained by Pearson's chi-squared test.

*Statistically significant results are in bold (*p* <0.05)

Supplementary Table S2 Genetic model analysis of *COL6A5* variants with lung cancer susceptibility among Chinese Han population

| SNP | Dominant^a^ | | Recessive^b^ | | Additive^c^ | |
| --- | --- | --- | --- | --- | --- | --- |
|  | OR (95% CI) | *p* value | OR (95% CI) | *p* value | OR (95% CI) | *p* value |
| rs77123808 | 0.93 (0.73 -1.20) | 0.582 | 0.87 (0.59 -1.27) | 0.465 | 0.93 (0.77-1.12) | 0.448 |
| rs10212241 | 0.99 (0.75 -1.29) | 0.911 | 1.03 (0.75 -1.41) | 0.866 | 1.00 (0.84 -1.20) | 0.983 |
| rs13062453 | **1.30 (1.01-1.68)** | **0.039*** | 1.05 (0.72-1.53) | 0.806 | 1.16 (0.97-1.40) | 0.105 |
| rs1497305 | 1.25 (0.97-1.60) | 0.086 | 0.66 (0.41-1.06) | 0.086 | 1.07 (0.87-1.30) | 0.530 |

SNP: Single nucleotide polymorphism; OR: Odds ratio; 95% CI: 95% confidence interval.

^a^Dominant model: BB *vs.* AB + AA; ^b^Recessive model: BB + AB *vs.* AA; ^c^Additive model: For each A allele increase (A: minor allele, B: wild allele).

The OR and 95% CI were calculated by logistic regression analysis.

*p* value was obtained by Wald test with adjustment for age and gender.

*Statistically significant results are in bold (*p* <0.05).

Supplementary Table S3 Genetic model analysis of *COL6A5* variants with lung cancer susceptibility after stratifying for age or gender

|  | SNP | Dominant^a^ | | Recessive^b^ | | Additive^c^ | |
| --- | --- | --- | --- | --- | --- | --- | --- |
|  |  | OR (95% CI) | *p* value | OR (95% CI) | *p* value | OR (95% CI) | *p* value |
| Age | > 61 | | | | | | |
|  | rs1497305 | 1.09 (0.77-1.56) | 0.619 | 0.56 (0.26-1.22) | 0.144 | 0.97 (0.73-1.30) | 0.863 |
|  | < 61 | | | | | | |
|  | rs1497305 | 1.40 (0.97-2.01) | 0.072 | 0.67 (0.37-1.25) | 0.208 | 1.12 (0.85-1.48) | 0.424 |
|  |  |  |  |  |  |  |  |
| Gender | Male | | | | | | |
|  | rs77123808 | 1.16 (0.86-1.57) | 0.330 | 0.71 (0.45-1.13) | 0.149 | 1.00 (0.80-1.25) | 0.987 |
|  | rs13062453 | 1.18 (0.88-1.59) | 0.275 | 1.02 (0.65-1.61) | 0.923 | 1.10 (0.88-1.37) | 0.399 |
|  | rs1497305 | 1.11 (0.82-1.50) | 0.496 | 0.61 (0.33-1.10) | 0.100 | 0.98 (0.77-1.25) | 0.895 |
|  | Female |  |  |  |  |  |  |
|  | rs77123808 | **0.58 (0.37-0.92)** | **0.021*** | 1.44 (0.70-2.97) | 0.323 | 0.80 (0.57-1.13) | 0.211 |
|  | rs13062453 | **1.68 (1.05-2.68)** | **0.030*** | 1.07 (0.55-2.09) | 0.837 | 1.33 (0.95-1.87) | 0.095 |
|  | rs1497305 | **1.59 (1.01-2.52)** | **0.046*** | 0.74 (0.34-1.61) | 0.450 | 1.23 (0.87-1.75) | 0.237 |

SNP: Single nucleotide polymorphism; OR: Odds ratio; 95% CI: 95% confidence interval.

^a^Dominant model: BB *vs.* AB + AA; ^b^Recessive model: BB + AB *vs.* AA; ^c^Additive model: For each A allele increase (A: minor allele, B: wild allele).

The OR and 95% CI were calculated by logistic regression analysis.

*p* value was obtained by Wald test with adjustment for age and gender.

*Statistically significant results are in bold (*p* <0.05).

Supplementary Table S4 Genetic model analysis of *COL6A5* variants with lung cancer susceptibility after stratifying for pathological type

| SNP | Dominant^a^ | | Recessive^b^ | | Additive^c^ | |
| --- | --- | --- | --- | --- | --- | --- |
|  | OR (95% CI) | *p* value | OR (95% CI) | *p* value | OR (95% CI) | *p* value |
| Lung Adenocarcinoma | | | | | | |
| rs13062453 | 1.41 (1.00-2.01) | 0.052 | 1.04 (0.63-1.74) | 0.872 | 1.21 (0.94-1.55) | 0.136 |
| rs1497305 | **1.51 (1.07-2.13)** | **0.018*** | **0.45 (0.22-0.96)** | **0.038*** | 1.14 (0.87-1.48) | 0.346 |
| Squamous Cell Carcinoma | | | | | | |
| rs13062453 | 1.16 (0.76-1.75) | 0.493 | 0.99 (0.52-1.86) | 0.965 | 1.08 (0.80-1.45) | 0.630 |
| rs1497305 | 1.21 (0.80-1.83) | 0.359 | 0.98 (0.47-2.05) | 0.960 | 1.12 (0.82-1.53) | 0.492 |

SNP: Single nucleotide polymorphism; OR: Odds ratio; 95% CI: 95% confidence interval.

^a^Dominant model: BB *vs.* AB + AA; ^b^Recessive model: BB + AB *vs.* AA; ^c^Additive model: For each A allele increase (A: minor allele, B: wild allele).

The OR and 95% CI were calculated by logistic regression analysis.

*p* value was obtained by Wald test with adjustment for age and gender.

*Statistically significant results are in bold (*p* <0.05).

Supplementary Table S5 Genetic model analysis of *COL6A5* variants with TNM staging of lung cancer among Chinese Han population

| SNP | Dominant^a^ | | Recessive^b^ | | Additive^c^ | |
| --- | --- | --- | --- | --- | --- | --- |
|  | OR (95% CI) | *p* value | OR (95% CI) | *p* value | OR (95% CI) | *p* value |
| rs1497305 | **0.57 (0.34-0.94)** | **0.028*** | **0.40 (0.18- 0.92)** | **0.031*** | **0.59 (0.40- 0.87)** | **0.008*** |

SNP: Single nucleotide polymorphism; OR: Odds ratio; 95% CI: 95% confidence interval.

^a^Dominant model: BB *vs.* AB + AA; ^b^Recessive model: BB + AB *vs.* AA; ^c^Additive model: For each A allele increase (A: minor allele, B: wild allele).

The OR and 95% CI were calculated by logistic regression analysis.

*p* value was obtained by Wald test with adjustment for age and gender.

*Statistically significant results are in bold (*p* <0.05).

Supplementary Table S6 Evaluation of the correlation between *COL6A5* variants and lymph node metastasis of lung cancer among Chinese Han population

| SNP | Allele/Genotype | Control N (%) | Case N (%) | OR (95% CI) | *p* value |
| --- | --- | --- | --- | --- | --- |
| rs77123808 | A | 111 (66.07%) | 292 (67.91%) | 1.00 | 0.667 |
|  | C | 57 (33.93%) | 138 (32.09%) | 0.92 (0.63-1.34) |  |
|  | AA | 33 (39.29%) | 96 (44.65%) | 1.00 |  |
|  | AC | 45 (53.57%) | 100 (46.51%) | 0.82 (0.48-1.41) | 0.478 |
|  | CC | 6 (7.14%) | 19 (8.84%) | 1.07 (0.39-2.94) | 0.889 |
| rs10212241 | C | 100 (59.52%) | 236 (54.88%) | 1.00 | 0.304 |
|  | T | 68 (40.48%) | 194 (45.12%) | 1.21 (0.84-1.74) |  |
|  | CC | 30 (35.71%) | 65 (30.23%) | 1.00 |  |
|  | CT | 40 (47.62%) | 106 (49.30%) | 1.24 (0.70 -2.19) | 0.459 |
|  | TT | 14 (16.67%) | 44 (20.47%) | 1.53 (0.72-3.23) | 0.266 |
| rs13062453 | G | 101 (60.12%) | 275 (64.25%) | 1.00 | 0.347 |
|  | A | 67 (39.88%) | 153 (35.75%) | 0.84 (0.58-1.21) |  |
|  | GG | 32 (38.10%) | 88 (41.12%) | 1.00 |  |
|  | GA | 37 (44.05%) | 99 (46.26%) | 0.90 (0.51 -1.57) | 0.703 |
|  | AA | 15 (17.86%) | 27 (12.62%) | 0.58 (0.27-1.24) | 0.158 |
| rs1497305 | G | 114 (67.86%) | 310 (72.43%) | 1.00 | 0.268 |
|  | A | 54 (32.14%) | 118 (27.57%) | 0.80 (0.55-1.18) |  |
|  | GG | 38 (45.24%) | 109 (50.93%) | 1.00 |  |
|  | GA | 38 (45.24%) | 92 (42.99%) | 0.83 (0.49 -1.41) | 0.486 |
|  | AA | 8 (9.52%) | 13 (6.07%) | 0.49 (0.19-1.31) | 0.155 |

SNP: Single nucleotide polymorphism; OR: Odds ratio; 95% CI: 95% confidence interval.

The OR and 95% CI were calculated by logistic regression analysis.

*p* value was obtained by Wald test with adjustment for age and gender.

Supplementary Table S7 Genetic model analysis of *COL6A5* variants with lymph node metastasis of lung cancer among Chinese Han population

| SNP | Dominant^a^ | | Recessive^b^ | | Additive^c^ | |
| --- | --- | --- | --- | --- | --- | --- |
|  | OR (95% CI) | *p* value | OR (95% CI) | *p* value | OR (95% CI) | *p* value |
| rs77123808 | 0.85 (0.51 - 1.44) | 0.554 | 1.19 (0.45 - 3.12) | 0.722 | 0.94 (0.62-1.41) | 0.757 |
| rs10212241 | 1.31 (0.77 - 2.25) | 0.319 | 1.34 (0.69 - 2.62) | 0.385 | 1.24 (0.86 -1.78) | 0.252 |
| rs13062453 | 0.81 (0.48- 1.37) | 0.422 | 0.61 (0.30-1.23) | 0.168 | 0.79 (0.54-1.14) | 0.204 |
| rs1497305 | 0.77 (0.46- 1.28) | 0.314 | 0.54 (0.21-1.38) | 0.199 | 0.76 (0.50- 1.14) | 0.179 |

SNP: Single nucleotide polymorphism; OR: Odds ratio; 95% CI: 95% confidence interval.

^a^Dominant model: BB *vs.* AB + AA; ^b^Recessive model: BB + AB *vs.* AA; ^c^Additive model: For each A allele increase (A: minor allele, B: wild allele).

The OR and 95% CI were calculated by logistic regression analysis.

*p* value was obtained by Wald test with adjustment for age and gender.
